# The Open-Air or Sanatorium Treatment of Pulmonary Tuberculosis

**Published:** 1910-03

**Authors:** 


**The Open-air or Sanatorium Treatment of Pulmonary Tuberculosis.** By F. RUFENACHT WALTERS, M.D., B.S. Lond. Pp. xiv, 323. London: Baillière, Tindall & Cox. 1909.

This excellent exposition of sanatorium life and work by one who is a thorough master of it cannot fail to be instructive to the reader. The first chapter concludes as follows:—

"There is no difficulty in preventing the spread of tuberculous infection if only people will take the trouble; moreover, if the bacillus gets into the body, no ill-effects follow unless the constitution has been undermined by unsatisfactory conditions of life, or has been enfeebled by a strong hereditary taint; so that the best safeguard of all is to improve the constitutional vigour by a return to more healthy ways of life."

How this is to be done effectually is fully disclosed in the twenty-seven chapters of Part I, and the 101 sections of Part II. It is intended that the book shall be of value not only to the doctor, but the intelligent patient, who needs guidance both in the sanatorium and after.

